# Dr. Ravel B. Parks

**Published:** 1897-12

**Authors:** 


Dr. Ravel B. Parks died suddenly at his home at Jamestown, N. Y., November 17, 1897, aged 48 years. He was stricken while in consultation in his office and lived but a few hours. It is supposed that heart disease was the cause of death.

Dr. Parks was one of the first graduating class of the Medical Department of Niagara University, having received his degree April 12, 1886. For a short time after graduation he practised his profession in Western Pennsylvania, and then removed to Falconer, Chautauqua County, New York, where he won honorable distinction as a physician. Recently he had taken up his residence in Jamestown, where he died.

